# Editorial: Bio-inspired legged robotics: design, sensing and control

**DOI:** 10.3389/frobt.2025.1600814

**Published:** 2025-07-03

**Authors:** Ting Zou

**Affiliations:** Department of Mechanical and Mechatronics Engineering, Memorial University of Newfoundland, St. John’s, NL, Canada

**Keywords:** legged robot, bio-inspired, mechanism design, sensing, control

## 1 Introduction

As a result of more than millions of years’ evolution, legged animals have showcased unrivaled sophisticated mobility, maneuverability and adaptation to complex environments, and have continuously inspired legged robot design. Bio-inspired legged robots have unique potential advantages in applications which require the transversal on rough terrain, exploration of unstructured environments and navigation in complex environments with obstacles that call for advanced maneuverability and control. In addition to the exceptional mobility, legged animals have established a complex sensing system with the environment that is significantly advantageous over the state-of-the-art robot sensation. With the impressive progress of artificial intelligence and sensing technologies in recent years, the bio-inspired legged robots have also experienced notable growth, accompanied by tremendous challenges though. This special collection aims to disseminate some of the latest advancements in the design, sensing and control of bio-inspired legged robots.

## 2 Overview of the papers in this special issue

The selected papers include theoretical and experimental research work on bicycle-inspired balance control method for quadruped robots (Hattori et al.); advancements of the understanding of the neural activity of robust robot locomotion control from computational neuroscience within deep reinforcement learning (Rush et al.); system design of a pneumatic-driven musculoskeletal bipedal robot with its sequential jumping experimental validation (Li et al.); investigations on the postural stabilization for a musculoskeletal robot during rapid and powerful hopping actions through the emulation of biarticular thigh muscles activation, along with experimental validation (Takahashi et al.); and the exploration of unknown environments using generalized autonomous mobile robots within simultaneous learning of the environments and robot transversality (Prágr et al.).

## 3 Conclusion

In conclusion, this Research Topic covers a broad range in the design, sensing and control of bio-inspired legged robots, and represents a significant step forward in the latest bio-inspired legged robot research. All papers in this issue present original, inspirational ideas, with a clear indication of problem formulation and methodologies, convincing experimental validations, potential applications, and proper paper organization. The achievements presented in these papers address some of the challenges and advance research on bio-inspired legged robot design, sensing and control. We hope this Research Topic will be helpful to enhance understanding and further research in this exciting field, and promote academic and industrial attention.

